# Use of 4CMenB vaccine in the control of an outbreak of serogroup B invasive meningococcal disease in an elderly care home, England, November 2023

**DOI:** 10.2807/1560-7917.ES.2025.30.16.2400673

**Published:** 2025-04-24

**Authors:** Emma J Heymer, Stephen A Clark, Helen Campbell, Sonia Ribeiro, Lloyd Walsh, Jay Lucidarme, Xilian Bai, Tom Irving, Anna Hoad, Jaime Morgan, Ray Borrow, Shamez N Ladhani

**Affiliations:** 1 Immunisation and Vaccine Preventable Diseases Division, UK Health Security Agency, Colindale, London, United Kingdom; 2 Meningococcal Reference Unit, UK Health Security Agency, Manchester Royal Infirmary, Manchester, United Kingdom; 3 South East Health Protection Team, UK Health Security Agency, United Kingdom; 4 Centre for Neonatal and Paediatric Infections (CNPI), St. George’s University of London (SGUL), London, United Kingdom

**Keywords:** MenB, outbreak, 4CMenB, vaccination, care home, infection control

## Abstract

In November 2023, a cluster of two invasive meningococcal disease (IMD) cases caused by serogroup B *Neisseria meningitidis* (MenB) occurred in elderly residents (≥ 70 years) of a dementia care home in England. An epidemiological investigation was conducted and public health actions, including infection control measures and antibiotic chemoprophylaxis, were implemented to prevent further cases. Nasopharyngeal swabbing before chemoprophylaxis identified three meningococcal carriers, including two carrying the outbreak strain, highlighting the importance of immediate antibiotic prophylaxis in such settings. Microbiological investigations showed that the outbreak strain belonged to the sequence type (ST)-9316 complex, potentially covered by the 4CMenB vaccine. Although 4CMenB is licensed for children and adults, there are no safety or reactogenicity data on use in older adults (≥ 65 years). Given the severity of IMD, residents (64–95 years) and staff (18–72 years) were offered 4CMenB for longer-term protection, with daily diary cards to monitor side effects. In total, 30 residents and 35 of 47 staff received the first dose, with completed diary cards for 26 residents and 32 staff. Twenty-six residents and 28 staff received the second dose, and all completed diary cards. Elderly residents reported fewer and less severe side effects after each dose than younger staff.

**Key public health message**

**What did you want to address in this study and why?**

Invasive meningococcal disease (IMD) is a rare but serious bacterial disease, which can require urgent hospital treatment, lead to life-changing disabilities or be fatal. We describe an outbreak by serogroup B *Neisseria meningitidis* (MenB) among two residents (≥ 70 years) in a care home in England in 2023. The 4CMenB vaccine, licenced in children and adults, was offered to elderly residents and staff as part of the response; adverse events were assessed.

**What have we learnt from this study?**

We found that residents (aged 64–95 years) were much less likely to experience adverse events after each dose of the 4CMenB vaccine compared with younger staff (aged 18–72 years), and we found a decreasing risk of adverse events with increasing age irrespective of resident/staff status. No additional IMD cases were identified after public health action, with the offer of antibiotics and vaccination, was undertaken.

**What are the implications of your findings for public health?**

Data on 4CMenB vaccine use in older adults are limited. We found that the 4CMenB vaccine was safe in older people, and there were no serious side effects, thus providing evidence to support its use in MenB care home outbreaks. Moreover, our findings support the current national guidelines in the United Kingdom on the use of the 4CMenB vaccine to control MenB outbreaks, including in care home settings.

## Background

*Neisseria meningitidis* is a human-specific commensal bacterium that is transmitted by the respiratory route and usually requires prolonged close contact. It is carried asymptomatically in the nasopharynx of 10% of the general population, with highest carriage rates in adolescents and young adults [1]. Rarely, the bacteria can cause invasive meningococcal disease (IMD), which commonly manifests as meningitis and/or septicaemia, and is associated with high morbidity and mortality. There are 12 known capsular groups based on their unique capsular polysaccharide composition, with serogroups A, B, C, W, X and Y causing nearly all IMD cases globally [2].

In 2022, 1,149 cases of IMD were reported from across 30 European countries, with a notification rate of 0.3 cases per 100 000 population [3]. In the United Kingdom (UK) [4], as in most European countries [3], serogroup B meningococci (MenB) are responsible for most IMD cases, with the highest annual incidence in infants (aged < 1 year) and toddlers (1–4 years), and a smaller peak in 15–24-year-olds [2]. In 2015, the UK became the first country to implement the four-component, protein-based 4CMenB vaccine for infants, resulting in a 75% reduction in invasive MenB disease in young children during the first 3 years of the programme [2]. MenB disease incidence was too low to make expansion of the programme to other age groups cost-effective. Also in 2015, in response to an outbreak of serogroup W (MenW) IMD, the UK implemented an adolescent MenACWY conjugate vaccine programme [5] offering protection against invasive disease and preventing carriage acquisition. By targeting the age group with the highest meningococcal carriage, this programme reduced MenW cases in vaccinated adolescents and provided indirect (herd) protection across the population [2].

From 2020, the COVID-19 pandemic restrictions led to large declines in respiratory infections, including a 73% reduction in all IMD cases in England [6]. As pandemic restrictions were lifted after July 2021, MenB cases in England increased but IMD caused by serogroups A, C, W and Y remained very low because of the ongoing adolescent MenACWY immunisation programme. Consequently, MenB remains the predominant serogroup in England, accounting for 88% (301/341) of IMD cases in the 2023/24 epidemiological year, including 74% (42/57) of IMD cases in those aged ≥ 65 years [4,7]. 

While almost all IMD cases are sporadic, clusters and outbreaks can occur, usually within households but also in other closed settings, such as nurseries, schools and residential halls of universities [8]. Management of IMD clusters may involve the use of mass antibiotic chemoprophylaxis and/or vaccination [9]. Elderly residential care homes are common settings for outbreaks of infectious diseases, especially those caused by respiratory and gastrointestinal pathogens, but IMD outbreaks are extremely rare, and we are not aware of any published reports of MenB outbreaks in a care home [10].

### Outbreak detection

In November 2023, two MenB IMD cases occurred one day apart in residents at a specialist dementia care home in England. A resident aged ≥ 70 years (Case A), who was well 2 h prior, became unresponsive with fever and low blood pressure. Case A was admitted to the hospital with suspected sepsis. The next day, a second resident in the same age group (Case B) was taken to the hospital with fever, rigors and dyspnoea. Invasive meningococcal disease was not initially suspected in either case. Two days after Case A was hospitalised, blood cultures became positive for *N. meningitidis* and the local UK Health Security Agency (UKHSA) Health Protection Team (HPT) was notified. The HPT initiated public health actions and advised the hospital to consider IMD in the differential diagnosis for Case B, who subsequently had a positive *N. meningitidis* blood culture 4 days after Case A was admitted. 

Here, we describe the public health response, including antibiotic chemoprophylaxis, pharyngeal swabbing, and the first use of 4CMenB vaccination in elderly care home residents, with daily diary cards to monitor adverse effects following vaccination.

## Methods

### Outbreak setting

The specialist dementia care home where the outbreak occurred can accommodate 32 residents, each in private single rooms with ensuite wet rooms. Bedrooms and two communal areas are located over four floors. The care home staff consisted of both permanent staff members and agency staff (temporary workers hired through an external agency) working across all floors who could all have had contact with both cases. Case A was receiving 1:1 daily care by different agency staff. Case B was socially active, spending time with other residents and staff.

### Case definition

The IMD case definition requires detection of *N. meningitidis* (culture/PCR) in a sterile site from a patient with clinical symptoms and signs consistent with meningitis, septicaemia or other invasive disease, thus qualifying both of these cases [9]. This IMD cluster of two cases caused by the same MenB strain, with onset within 28 days in a care home setting, met the outbreak definition described in UK guidance on public health management of meningococcal disease [9].

### Microbiological investigations

Clinical specimens, including blood cultures, from the two patients with suspected sepsis were processed at the local National Health Service (NHS) laboratory. *Neisseria meningitidis* cultures are routinely submitted to the UKHSA Meningococcal Reference Unit (MRU) for characterisation including serogrouping by dot blot ELISA [11], antibiotic susceptibility testing using Etest gradient strips (bioMérieux), whole genome sequencing using Illumina [12], and Meningococcal Antigen Typing System (MATS) testing to determine potential protection by the 4CMenB (Bexsero, GSK Biologicals, Belgium) vaccine [13]. 

Relatedness of the infecting strains was investigated using the Genome Comparator tool (Scheme: *N. meningitidis* cgMLST V3; PubMLST.org) [14]. Distances were visualised as a NeighborNet network using SplitsTree4 [15].

Pharyngeal swabs were processed at the local NHS laboratory using selective gonococcal agar supplemented with vancomycin, colistin, amphotericin and trimethoprim. Positive meningococcal isolates were submitted to the MRU for characterisation. Concurrent viral throat swabs were also taken from residents and staff experiencing respiratory symptoms to identify any respiratory viral transmission that might have been propagating the IMD outbreak [16].

### Incident management structure

The outbreak response was coordinated through an incident management team (IMT), led by the UK Health Security Agency (UKHSA) local Health Protection Team (HPT) managing public health action for the cases. The IMT included laboratory staff from the UKHSA Meningococcal Reference Unit (MRU), epidemiologists and clinicians from the National UKHSA Immunisation Division, and members of the Regional UKHSA Communications Team, alongside the care home manager, local GP surgery, local council and those responsible for planning local health services on the NHS local integrated care board. The role of the IMT was to coordinate the public health investigation and agree on appropriate public health actions to prevent further IMD cases in the care home. Additional steps to better understand transmission in the care home and an assessment of the acceptability, tolerability and reactogenicity of 4CMenB were also discussed. The IMT met twice, with the first meeting held on the day that the second IMD case was confirmed.

### Chemoprophylaxis, vaccination and recording adverse events

Antibiotic chemoprophylaxis, in the form of a single dose of ciprofloxacin, should be offered to anyone who has had prolonged close contact with a case in the 7 days before disease onset. For clusters in a residential setting such as this, where a clear high-risk group such as residents and staff is defined, antibiotic chemoprophylaxis should be offered to everyone in that group. 

National guidelines recommend the use of the 4CMenB vaccine to control MenB outbreaks in the UK, including in care home settings; two doses should be offered with a 4-week interval [9]. 

Because reactogenicity or safety data were lacking on 4CMenB vaccine use in older adults, we developed a standard diary card to record side effects, i.e. adverse events (ADRs) such as pain and/or swelling at the injection site, for 7 days after each vaccine dose. The diary card for recording side effects is provided as Supplementary Figure S1. The diary cards included a pain scale for side effects in three grades: Grade 1 - no disruption to normal daily activities, Grade 2 - enough to reduce or affect normal daily activity to some degree and Grade 3 - side effect reduced or affected normal daily activity considerably for at least 24 h. Staff caring for residents who were vaccinated completed the diary card on their behalf. For comparison, diary cards were also completed by staff who received the vaccine. 

### Statistical analysis

Proportions of residents and staff reporting ADRs were compared using the chi-square test in Stata version 18.0 (StatCorp). Data were also analysed by age group: 18–39, 40–59, 60–79 and ≥ 80 years.

## Results

**Figure 1:**
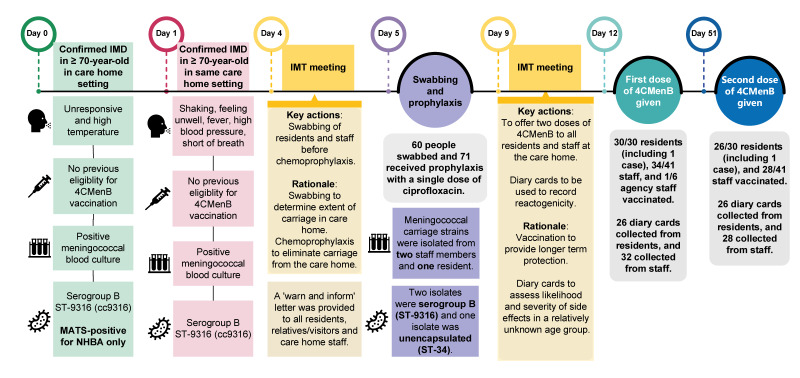
Timeline of events and public health actions following an invasive meningococcal disease outbreak in a care home, England, November 2023 (n = 2 cases)

IMD: invasive meningococcal disease; IMT: incident management team; NHBA: neisserial heparin-binding antigen.

Case A experienced symptom onset in November 2023, with Case B experiencing onset the following day. Both care home resident cases were ≥ 70 years and were admitted to the hospital upon onset of symptoms. The next day, blood cultures for Case A became positive for *N. meningitidis*. Two days later, when Case B had a positive *N. meningitidis* blood culture, although serogroups were not yet determined, an outbreak was declared by the HPT. As per national public health guidance [9], an IMT was established to initiate a public health response and manage the outbreak. The outbreak timeline is given in Figure 1. 

The timeframe is based on the interval between day 0 (onset of Case A) and the activity indicated, using time in days. Antibiotic chemoprophylaxis, in the form of a single dose of ciprofloxacin, was offered to all staff and residents. Vaccination with 4CMenB: 30 residents vaccinated with the first dose included 1 case and 29 non-cases, and 26 residents vaccinated with the second dose also included 1 case and 25 non-cases. The diary card for recording side effects of vaccination is provided as Supplementary Figure S1.

At the start of the outbreak, there were a total of 31 residents including the two IMD cases (median age: 83 years; range: 64–95) and 47 staff comprised of 41 permanent staff and 6 agency staff (median age: 45 years; range: 18–72). Both IMD cases survived and were discharged from the hospital. After implementation of infection prevention and control measures and antibiotic chemoprophylaxis, there were no additional IMD cases in the setting.

### Outbreak control measures

A ‘warn and inform’ letter prepared by the UKHSA HPT was provided immediately following the first IMT meeting to all residents and relatives/visitors alongside care home staff, explaining the importance of infection prevention, the control measures being implemented, and advising against visiting the care home if unwell. Local hospital emergency departments, microbiologists and general practitioners (GPs) were alerted to the IMD cluster and asked to be vigilant for further potential cases. Care home staff were asked to wear personal protective equipment (PPE) including gloves, aprons and facemasks, and good hand hygiene guidance was reiterated.

Voluntary pharyngeal swabbing was offered to all residents and staff to assess meningococcal carriage in the care home prior to taking chemoprophylaxis. Informed consent was obtained for all individuals. Those providing a pharyngeal swab were reassured that the prophylactic antibiotic dose would eliminate meningococcal carriage irrespective of the pharyngeal swab culture results. Pharyngeal swab samples were provided by all but one of the 29 non-hospitalised residents, 30 permanent staff and two agency staff, with three samples positive for *N. meningitidis*. Antibiotic chemoprophylaxis for close contacts, in this case care home residents and staff, eliminates carriage, thereby reducing onward transmission and offers short-term protection against development of invasive disease. As part of the outbreak response, all residents and staff were offered chemoprophylaxis with a single ciprofloxacin dose on the fifth day after the Case A was hospitalised [11]. Chemoprophylaxis was accepted by all 29 other residents, 37 of 41 permanent staff and 5 of 6 agency staff.

### Microbiological investigations

Culture isolates from both cases (isolate IDs: 23MN0386 and 23MN0387) were characterised as MenB, sequence type (ST)-9316 complex. Upon MATS analysis, 23MN0386 tested strongly positive for neisserial heparin-binding antigen (NHBA) (relative potency (RP) = 1.329; positive bactericidal threshold (PBT) > 0.294) indicating the strain was covered by the 4CMenB vaccine.

**Figure 2:**
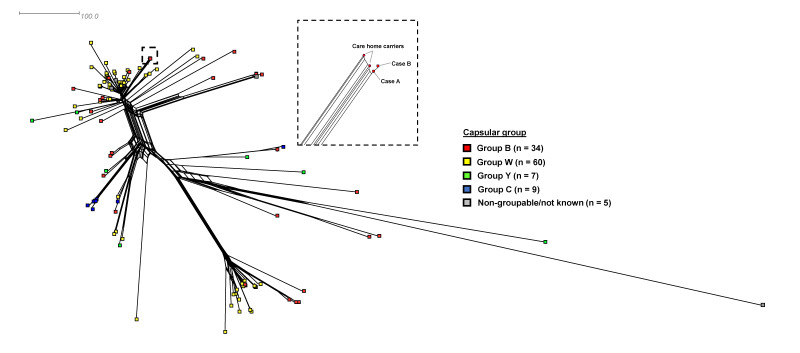
A core genome comparison of meningococcal ST-9316 complex isolates including care home carriage and invasive isolates, England, November 2023 (n = 115)

Additionally, two carriage isolates (one resident, one staff) were identified as MenB:ST-9316 and were indistinguishable from the invasive isolates from the two cases using core genome MLST analysis. When compared with ST-9316 genomes on PubMLST.org, they formed an exclusive monophyletic group with no allelic differences among 1,344 loci (Figure 2) [17]. The third pharyngeal isolate from another staff member was distinct (non-groupable (capsular null), ST-34 (ST-32 complex; cc32)).

All ST-9316 complex isolate genomes on the PubMLST.org Neisseria database (accessed: 2 April 2024) were included (n = 115). Distances were visualised as a NeighborNet network using SplitsTree4 [15]. This included isolates from multiple countries (including the United Kingdom, France, Germany and Czechia, among others) and years (2010–23). The collection included invasive (n = 102) and carriage isolates (n = 13). Isolates are highlighted with coloured boxes to show capsular group distribution. The non-ST-9316 carriage strain isolated from the care home (ST-34) was included on the diagram as a reference.

Simultaneous viral swabbing was performed in eight individuals with respiratory symptoms, including one resident positive for *N. meningitidis* carriage. We identified low levels of respiratory syncytial virus (RSV) RNA in two of the eight individuals, neither of whom were identified as carriers of *N. meningitidis*. All symptomatic individuals recovered uneventfully after a few days.

### Meningococcal vaccination

Since a single dose of ciprofloxacin provides only short-term protection against IMD among contacts of confirmed cases [18], 4CMenB vaccination was offered for longer-term protection, as per UK national guidance [9]. 4CMenB was offered to all residents and staff in the care home and was not limited to those who accepted antibiotic prophylaxis.

The vaccine was administered as a two-dose schedule with an interval of at least 4 weeks between doses [10]. The first dose of vaccine was administered 12 days after Case A’s symptom onset date. In total, 30 residents (the 29 non-cases and Case B returned from the hospital), 34 of 41 permanent staff and 1 of 6 agency staff received the first 4CMenB dose, with 26 residents and 28 staff (83% of those receiving the first dose) receiving the second dose 39 days later. Case A remained in the hospital and did not receive the vaccine.

Completed diary cards were obtained for 26 residents and 32 staff after the first vaccine dose. Adverse events were reported for only 5 of 26 residents compared with 28 of 32 staff (Table 1), and for 5 of 26 and 22 of 28, respectively, after the second dose. Of note, four were the same after both Dose 1 and Dose 2; one resident reported ADRs only after Dose 1 and another resident reported ADRs only after Dose 2. Staff were significantly more likely than residents to experience at least one adverse event following 4CMenB vaccination (chi-square = 27.2, p value < 0.001).

**Table 1:** Adverse events following 4CMenB vaccination among care home residents and staff during an outbreak of invasive meningococcal disease, England, November 2023 (n = 65)

Adverse events as reported on diary cards	Number of residents		Number of staff	
	Dose 1	Dose 2	Dose 1	Dose 2
Total vaccinated	30	26	35	28
Completed diary cards	26	26	32	28
Reported adverse events				
No	21	21	4	6
Yes	5	5	28	22
Pain at injection site^a^				
Any pain	5	5	28	22
Grade 1	4	5	17	13
Grade 2	1	0	6	2
Grade 3	0	0	1	1
Not graded	0	0	4	6
Swelling at injection site^a^				
Any swelling	5	5	24	19
Grade 1	4	5	15	14
Grade 2	1	0	5	0
Not graded	0	0	4	5
Other symptoms				
Itching at injection site	0	0	2	1
Feeling hot/feverish	0	0	4	2
Confirmed fever > 38 °C	0	0	1	1
Tiredness	0	0	8	4
Headache	0	0	6	3
Vomiting	0	0	1	1
Diarrhoea	0	0	1	0
Loss of appetite	0	0	3	1
Rash	0	0	1	0
Muscle aches and pains	0	0	7	4
Bone/joint aches and pains	0	0	6	3
Itching all over body	0	0	2	0

MenB: serogroup B meningococci.

^a^ Grade 1: no disruption to normal daily activities; Grade 2: enough to reduce or affect normal daily activity to some degree; Grade 3: reduced or affected normal daily activity considerably for at least 24 h.

The diary card for recording side effects is provided as Supplementary Figure S1.

**Figure 3:**
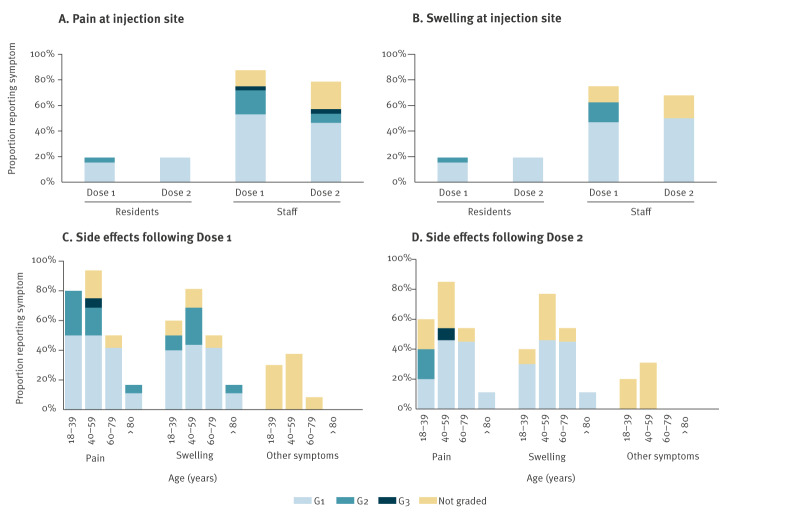
Proportion of care home residents and staff that completed a daily diary card who reported each symptom following 4CMenB vaccination during an outbreak of invasive meningococcal disease, England, November 2023 (n = 58)

Five residents reported pain and swelling at the injection site after each dose. After Dose 1, four residents reported these adverse events at Grade 1, while one resident reported Grade 2. After Dose 2, all five reported pain and swelling at Grade 1 (Table 1, Figure 3).

Graphical representations of residents and staff who reported pain (A) or swelling (B) at the site of the injection, by dose and grade of symptom. For residents and staff for whom date of birth was recorded (n = 56), side effects after 4CMenB vaccination including pain or swelling at injection sites, or other symptoms are shown by age group after Dose 1 (C) and Dose 2 (D).

The diary card for recording side effects is provided as Supplementary Figure S1.

Of the 28 of 32 staff members who reported any adverse events after Dose 1, all 28 reported pain at the injection site (with n = 7 reporting Grade 2 or above) and 24 of 28 reported swelling at the injection site (with n = 5 reporting Grade 2 or above). Collectively, at least one staff member reported experiencing every other solicited adverse event after the first dose. Eight staff members reported tiredness, six reported headaches, and six reported bone/joint aches and pains. One staff member who experienced all but one of the solicited adverse events after the first dose was advised by clinicians to decline the second dose.

After Dose 2, 22 of 28 staff members reported at least one solicited adverse event, including pain (22/22) or swelling (19/22) at the injection site. Six staff members experienced other adverse events, and collectively reported 9 of the other 12 solicited adverse events.

There was an overlap between the staff and residents’ ages, with three residents being younger than at least one staff member. Findings by age group showed that the proportion of individuals experiencing pain or swelling at the injection site or any other adverse event, was highest among 40–59-year-olds, followed by those aged 18–39 years (Table 2, Figure 3). Those aged ≥ 80 years were least likely to experience any adverse event after vaccination (3/18) and only reported Grade 1 pain or swelling at the injection site except one resident who reported Grade 2 after Dose 1 only. No residents or staff members experienced serious adverse events or hospitalisations after vaccination.

**Table 2:** Adverse events following 4CMenB vaccination by dose and age group among care home residents and staff during an outbreak of invasive meningococcal disease, England, November 2023 (n = 56)

Adverse events as reported on diary cards	Number of residents and staff^a^							
	Dose 1				Dose 2			
	18–39 years	40–59 years	60–79 years	≥ 80 years	18–39 years	40–59 years	60–79 years	≥ 80 years
Completed diary cards	10	16	12	18	10	13	11	18
Reported adverse events								
No	2	1	6	15	4	2	5	16
Yes	8	15	6	3	6	11	6	2
Pain at injection site^b^								
Any pain	8	15	6	3	6	11	6	2
Grade 1	5	8	5	2	2	6	5	2
Grade 2	3	3	0	1	2	0	0	0
Grade 3	0	1	0	0	0	1	0	0
Not graded	0	3	1	0	2	4	1	0
Swelling at injection site^b^								
Any swelling	6	13	6	3	4	10	6	2
Grade 1	4	7	5	2	3	6	5	2
Grade 2	1	4	0	1	0	0	0	0
Not graded	1	2	1	0	1	4	1	0
Other symptoms								
≥ 1 symptom	3	6	1	0	2	4	0	0

^a^ Ages were not available for two staff members who completed the forms but staff/resident status was available for all.

^b^ Grade 1: no disruption to normal daily activities; Grade 2: enough to reduce or affect normal daily activity to some degree; Grade 3: reduced or affected normal daily activity considerably for at least 24 h.

## Discussion

To our knowledge, this is the first MenB outbreak in a care home where 4CMenB was offered to older adults (≥ 65 years) for individual protection. This was done despite the absence of safety or reactogenicity data for use of the vaccine in this vulnerable population. The decision to offer 4CMenB to all residents and staff was made because of the severity of IMD and high case-fatality rates in older adults, i.e. as high as 33% in those aged over 80 years compared with 15% in younger adults and 9% in infants [19]. Consequently, the IMT decided that any potential risks of vaccination were far outweighed by the benefits of individual medium-to-long-term protection against IMD in the context of an outbreak in a closed setting. Additionally, 4CMenB has already been given safely to millions of younger adults, adolescents and children worldwide [20], and our previous experience with 4CMenB indicated that the reactogenicity and other adverse events associated with vaccination declined with increasing age in adults.

Given the lack of data, we asked residents to complete a daily diary card after each dose. For comparison, we also asked vaccinated staff to complete this task. We found that residents were significantly less likely to experience adverse events after each vaccine dose compared with staff and observed a lower risk of adverse events among older participants irrespective of resident/staff status. It is, however, important to acknowledge that elderly residents, especially those with dementia, may have been less able to express or communicate their symptoms than staff.

Our observation that around 80% of care home staff experienced at least one adverse event after vaccination is consistent with a published report that over 75% of adults in special situations with a mean age of 52.5 years reported local pain at the injection site [21]. Similarly, in another study where 4CMenB was given to adults at increased risk for occupational exposure to meningococci, pain at the injection site was reported by all participants [22,23]. Since 4CMenB is rarely indicated or used in older adults, there are limited data on safety, tolerability or immunogenicity in this vulnerable group. An early investigation into 4CMenB use only included adults aged up to 40 years [24], and an Italian post-marketing study collected adverse effects following 4CMenB vaccination up to age 32 years [25]. Similarly, a study from the United States assessing almost 2,000 reports of adverse events included only 47 reports among those aged 26 years or older [26], and in a German post-marketing study, there were only three people aged 60 years and over, with a maximum age of 69 years [27]. To date, published data indicate that adverse events are less common in adults than young children, and that the 4CMenB vaccine is safe for use in all age groups [28]. However, additional studies to expand the limited safety data collected for older adults in our outbreak investigation would be beneficial to inform future public health recommendations.

This MenB care home outbreak provided a unique opportunity to assess adverse events after 4CMenB vaccination in elderly residents compared with younger healthy adult staff. Our findings are reassuring and indicate that 4CMenB can be safely used in older adults for personal protection against MenB IMD. The lower reactogenicity in older adults, however, may be indicative of a lower immune response to vaccination, most likely explained by immune senescence and underlying co-morbidities that may impair vaccine responses in this vulnerable population, as well as reduced ability among elderly residents with dementia to report adverse events. We offered vaccination as part of the public health response to a care home outbreak and, therefore, did not collect blood samples to assess immunogenicity in residents or staff. Future studies of vaccine responses in older age groups would be valuable to assess the level of protection provided by vaccination.

The collection of pharyngeal bacterial swabs from residents and staff provided useful insight into meningococcal carriage and transmission in care homes, especially given that adolescents are the main nasopharyngeal carriers and sources of meningococci transmission. The identification of two carriers of the responsible MenB strain, alongside the two confirmed cases, similar to a previously reported MenW care home cluster [10], indicates that the outbreak strain was circulating within the care home. This highlights the critical importance of urgent antibiotic chemoprophylaxis to all relevant contacts as soon as a meningococcal outbreak is identified in a closed setting.

Previous care home outbreak investigations have often identified bacterial co-transmission with viral infections [29]. In the reported outbreak, eight individuals were experiencing respiratory symptoms when swabbed, including two with confirmed RSV infection. This demonstrates that even a single case of a serious bacterial infection could be demonstrative of co-transmission of other viral infections. This highlights the importance of early infection control measures, even after a single case of a serious bacterial infection in a care home, as multiple pathogens may be transmitting through the vulnerable population in tandem. Had influenza been identified among the symptomatic individuals, staff and residents would also have been offered antivirals as per national guidelines [30], especially since influenza infections can also increase IMD risk [16].

Whole genome sequencing of the meningococcal isolates provided invaluable information on the outbreak strain and, with MATS, supported the decision to use 4CMenB as part of the outbreak response. The responsible strain belonged to the ST-9316 complex, which has caused IMD across many European countries in recent years, including MenW outbreaks in France [31]. Core genome analysis of the outbreak strain alongside other European cc9316 strains demonstrated a wide distribution of serogroups including B, W and Y, indicating a propensity for capsular switching. In this outbreak, the strain was covered by the NHBA component of 4CMenB (peptide 243) with a MATS RP of 1.329 (PBT > 0.294) [32]. Alleles for this NHBA variant were harboured by most other ST-9316 complex strains (data not shown). The strain had fHbp peptide 321 (variant 1), but this was not covered according to MATS (RP = 0.007; PBT > 0.012) and neither was the mismatched PorA (P1.5–2,10–1) or NadA (peptide 21 variant NadA-4/5) [33].

Our findings support current national guidelines on the use of 4CMenB to control MenB outbreaks, even in care home settings where data on 4CMenB use in older adults are limited [9]. We found that both doses of 4CMenB were less reactogenic in older compared with younger adults, and there were no serious adverse events. The immunogenicity of two doses in older adults, however, is not known and future studies or outbreak responses should consider collecting pre- and post-vaccination blood samples for immunogenicity studies after obtaining appropriate research ethics approval and informed consent. Future studies and outbreak responses could also seek to record adverse events following 4CMenB in elderly populations without dementia, to confirm that the lower reactogenicity in the elderly was not related to cognitive impairment.

Because the cluster occurred during a timeframe approaching the Christmas holiday period and a minimum 4-week gap between doses of 4CMenB vaccine is recommended, one limitation of this investigation was an additional delay in administering the second dose of the vaccine to staff and residents. Not all staff and residents received one or both vaccine doses, but no information is available on reasons for declining. Not everyone who accepted vaccination completed the diary cards, so details of side effects are limited to individuals who participated. Additionally, care home residents’ diary cards were completed by proxy and therefore some side effects may have been missed.

## Conclusions

Here, we have reported the first use of 4CMenB to help control a MenB outbreak in a care home. By systematically collecting data on symptoms after each vaccine dose, we have shown that older adults are less likely to report adverse events after 4CMenB, with similar prevalence of reactions following each dose, thus providing support for its use in any future MenB care home outbreaks. The identification of asymptomatic pharyngeal carriage of the meningococcal outbreak strain highlights the critical importance of offering antibiotic chemoprophylaxis as soon as an outbreak is identified to prevent further cases and interrupt transmission to others in the care home.

## Supplementary Data

272000 Supplement
