# Supplementary material for: Use of 4CMenB vaccine in the control of an outbreak of serogroup B invasive meningococcal disease in an elderly care home, England, November 2023
Source: Euro Surveill. 2025 Apr 24;30(16):2400673. doi: 10.2807/1560-7917.ES.2025.30.16.2400673 (PMC12023728; doi:10.2807/1560-7917.ES.2025.30.16.2400673)
Supplement: Supplement [file 24-00673_HEYMER_Supplement.pdf]

This supplementary material is hosted by Eurosurveillance as supporting information alongside the article [Use of 4CMenB vaccine in the control of an outbreak of serogroup B invasive meningococcal disease in an elderly care home, England, November 2023] on behalf of the authors who remain responsible for the accuracy and appropriateness of the content. The same standards for ethics, copyright, attributions and permissions as for the article apply. Eurosurveillance is not responsible for the maintenance of any links or email addresses provided therein.

Supplementary Figure S1: Diary card for residents and staff to record side effects and their severity after each dose of the vaccine

Men B vaccination – side effect data collection form

Name of vaccine recipient: **Staff or Resident:**

Date of birth: **Date vaccine dose 2 received:**

Injection site: **Left arm ☐** **Right arm ☐**

Please complete all the rows in the table below, using the following GRADE for symptoms:

1: No disruption to normal daily activities
2. Enough to reduce or affect normal daily activity to some degree
3. Reduced or affected normal daily activity considerably for at least 24 hours

	Occurred	Did not occur	Unable to assess	Grade if present (1, 2 or 3)	Start date	End date
Pain at the site of injection	☐	☐	☐			
Swelling at the site of injection	☐	☐	☐			
Itching at the site of injection	☐	☐	☐			
Feeling hot/fever-ish	☐	☐	☐			
Confirmed fever >38 °C. Please state temperature	☐	☐	☐			
Tiredness (more than normal)	☐	☐	☐			
Headache (more than normal)	☐	☐	☐			
Vomiting	☐	☐	☐			
Diarrhoea	☐	☐	☐			
Loss of appetite (more than normal)	☐	☐	☐			
Rash	☐	☐	☐			
Muscle aches and pains (more than normal)	☐	☐	☐			
Bone/joint aches and pains (more than normal)	☐	☐	☐			
Itching all over body	☐	☐	☐			
Other (specify below)	☐	☐	☐			
